# Occurrence and Diversity of CRISPR-Cas Systems in the Genus *Bifidobacterium*

**DOI:** 10.1371/journal.pone.0133661

**Published:** 2015-07-31

**Authors:** Alexandra E. Briner, Gabriele Andrea Lugli, Christian Milani, Sabrina Duranti, Francesca Turroni, Miguel Gueimonde, Abelardo Margolles, Douwe van Sinderen, Marco Ventura, Rodolphe Barrangou

**Affiliations:** 1 Department of Food, Bioprocessing and Nutrition Sciences, North Carolina State University, Raleigh, North Carolina, United States of America; 2 Laboratory of Probiogenomics, Department of Life Sciences, University of Parma, Italy; 3 School of Microbiology and Alimentary Pharmabiotic Centre, University College Cork, Western Road, Cork, Ireland; 4 Dairy Research Institute of Asturias, Spanish National Research Council (IPLA-CSIC), Villaviciosa, Asturias, Spain; University of Ulm, GERMANY

## Abstract

CRISPR-Cas systems constitute adaptive immune systems for antiviral defense in bacteria. We investigated the occurrence and diversity of CRISPR-Cas systems in 48 *Bifidobacterium* genomes to gain insights into the diversity and co-evolution of CRISPR-Cas systems within the genus and investigate CRISPR spacer content. We identified the elements necessary for the successful targeting and inference of foreign DNA in select Type II CRISPR-Cas systems, including the tracrRNA and target PAM sequence. *Bifidobacterium* species have a very high frequency of CRISPR-Cas occurrence (77%, 37 of 48). We found that many *Bifidobacterium* species have unusually large and diverse CRISPR-Cas systems that contain spacer sequences showing homology to foreign genetic elements like prophages. A large number of CRISPR spacers in bifidobacteria show perfect homology to prophage sequences harbored in the chromosomes of other species of *Bifidobacterium*, including some spacers that self-target the chromosome. A correlation was observed between strains that lacked CRISPR-Cas systems and the number of times prophages in that chromosome were targeted by other CRISPR spacers. The presence of prophage-targeting CRISPR spacers and prophage content may shed light on evolutionary processes and strain divergence. Finally, elements of Type II CRISPR-Cas systems, including the tracrRNA and crRNAs, set the stage for the development of genome editing and genetic engineering tools.

## Introduction

Clustered regularly interspaced short palindromic repeats (CRISPR) with CRISPR-associated (*cas*) genes constitute the CRISPR-Cas adaptive immune systems in bacteria and archaea [1]. CRISPR loci are identified in genomes by locating regions of conserved repeats interspersed by short variable sequences, spacers, that are adjacent to *cas* genes. The immune function of the systems is carried out by Cas proteins in three steps: first, the *acquisition* of DNA from an invader is stored between conserved repeats; next, the entire repeat-spacer array is transcribed into a single RNA transcript that must be processed into individual small interfering units during the *expression* phase; and finally, during *interference*, the guide RNA direct Cas proteins to its target and cleaves foreign invaders in a highly sequence-specific manner upon reinfection [2–6]. Repeat-spacer arrays record the immunization timeline of an organism as spacer sequences, which are always added to the array at the leader end; this can provide a unique, hypervariable locus that can be used to genotype organisms so as to provide insights into their phylogenetic divergence and relatedness to other strains [7]. Identifying the source of the spacer sequences, called the protospacer sequence, can tell a story of host-prey dynamics in environments where phage and plasmid intrusions are common and often lethal [8]. Several early CRISPR metagenomic and ecological studies have investigated the co-evolutionary effects of phage predation and CRISPR-acquired defense on both the host bacterium and predatory mobile genetic material [8–12]. Iterative phage infection and consequent mutations to escape CRISPR targeting drive phage genome evolution as well as host strain evolution through adaptation and acquisition of new immunity-conferring spacers at CRISPR loci [1,8–12].

Three types of CRISPR-Cas systems with distinct locus architecture and Cas effector proteins have been described [13]. While all have the universal *cas1* gene, each main type has a signature gene used to classify CRISPR loci into types: *cas3* for Type I, *cas9* for Type II, and *cas10* for Type III. Type I and Type III systems carry out the interference stage of immunity using a large CRISPR associated complex for anti-viral defense (Cascade) protein complex composed of several Cas protein subunits [3,5]. In contrast, Type II systems only require a single endonuclease, Cas9, to effectively target and cleave foreign DNA [14–16]. In all three systems, RNA molecules, called CRISPR RNAs (crRNAs), that contain a partial CRIPSR repeat and partial spacer guide the Cas proteins to their DNA targets. In Type I systems, there is only a single crRNA to direct the CASCADE complex, while Type II systems require an additional *trans*-activating CRISPR RNA (tracrRNA) bound to the crRNA to form a crRNA:tracrRNA duplex [2–6]. Recently, the Cas9 machinery has been developed into a sequence-specific DNA-targeting and cleaving tool through re-programming of the native protein and crRNA:tracrRNA duplex, revolutionizing genetic engineering [17–19].

Bifidobacteria have become increasingly studied as their potential to positively affect human host health becomes better characterized [20–21]. Through their interactions with the human gastrointestinal tract (GIT), bifidobacteria may help fortify the intestinal barrier, modulate immune responses, and competitively exclude pathogens [20–21]. Bifidobacteria have characteristically small genomes ranging in size from 2.0 to 2.8 Mbp [22]; the small size of bifidobacterial genomes is hypothesized to be caused by genome decay as dispensable genes are lost driving adaption to a specific ecological niche [23–24]. While many intra-species studies have thoroughly investigated genomic differences within single species like *Bifidobacterium animalis* [25], *Bifidobacterium dentium* [26], *Bifidobacterium bifidum* [27],as well as *Bifidobacterium breve* and *Bifidobacterium longum* [28], only a few studies have investigated differentiating features of species across the genus [23, 29].

While bifidobacterial genomes have been studied fairly extensively, little research has focused on elucidating the role that parasitic phages and prophages have played in shaping the fitness of different *Bifidobacterium* species. Previous work by Ventura et al. investigated the prevalence of prophages in bifidobacteria and was the first study to propose that the interplay between bifidobacteria and phages drives coevolution of both the phage and host as evidenced by the prevalence of prophages and CRISPR-Cas systems [20,30]. Overall, 22 prophage-like elements have been characterized in the genus *Bifidobacterium*, occurring in nine strains representing six species that include: *B*. *dentium*, *B longum*, *B*. *bifidum B*. *animalis* and *Bifidobacterium adolescentis* [20, 30–31]. Several studies have suggested that prophages in bifidobacteria are a major source of lateral gene transfer and driver of population adaptation to environmental niches [20, 30–31]. Prophage integration is a proposed mechanism for host adaptation as some prophage-elements include genes for increased host viability like polyketide biosynthesis, type I restriction modification systems, and retro-type reverse transcriptase [20].

In this study, we aimed to investigate the prevalence and diversity of CRISPR-Cas systems in 48 *Bifidobacterium* genomes in an attempt to gain insights into the phylogenetic history and biological importance of these systems. First, we identified and characterized repeat-spacer arrays and *cas* genes. Next, we compared the different systems using conserved elements to look for system relatedness and divergence. Spacers were further analyzed to identify homology to foreign DNA sequences in genomic and metagenomic data to determine potential functional roles these immune systems might play. Further characterization of Type II CRISPR-Cas systems were performed to identify elements necessary for interference, including the non-coding tracrRNA and target PAM sequence, for select systems.

## Materials and Methods

### *In silico* analyses

The genomes used were previously described by Milani et al. [19] (Table 1). CRISPR analyses were performed using CRISPRfinder followed by manual curation to identify and assemble repeat-spacer arrays [32]. BLAST [33] was used to identify Cas proteins and confirm subtype designation [13]. SnapGene (GSL Biotech) was used to annotate *cas* genes and generate the operon layout. The Cas1 alignment was performed using the MUSCLE alignment algorithm; the consensus tree was constructed in MEGA version 6 [34] using the UPGMA Jukes-Cantor model calculating bootstrap values with 500 samples. Protospacers were identified using BLASTn against publically available metagenomic datasets and a locally created bifidobacterium database. A “strong” protospacer match was considered when two examined sequences exhibited more than 90% identity over 90% of the spacer sequence length. Additional analyses were performed to detect similarity between CRISPR spacers and prophage sequences located within the bifidobacterium genomes used in this study. Once potential protospacers were identified, the flanking regions were aligned by hand and analyzed by WebLogo to determine the protospacer adjacent motif (PAM) [35]. The tracrRNA sequences for *Bifidobacterium bombi*, *Bifidobacterium merycicum*, and *B*. *bifidum* were predicted manually using BLAST to identify anti-repeat region in the respective genomes per the modules previously defined by Briner et al. [36]. NUPACK was used to generate RNA folding prediction images [37].

**Table 1:** Occurrence of CRISPR-Cas systems in bifidobacteria.

*Bifidobacterium* Species	Strain	System Type	CRISPR Repeat Sequence	RepeatLength	Numberof Repeats	*cas1*	*cas3*	*cas9*	*cas10*
*B*. *actinocoloniiforme*	DSM 22766	I-E	GTGTTCCCCGCATGCGCGGGGATGATCCC	29	81	Y	Y		
*B*. *adolescentis*	ATCC 15703	I-C	GTCGCTCTCCTTACGGAGAGCATGGATTGAAAT	33	86	Y	Y		
*B*. *angulatum*	LMG 11039	I-E	GTGTTCCCCGCACACGCGGGGATGATCCC	29	172	Y	Y		
*B*. *animalis subsp*. *animalis*	ATCC 25527	I-E	GTTTGCCCCGCACAGGCGGGGATGATCCG	29	32	Y	Y		
*B*. *animalis subsp*. *lactis*	DSM 10140	I-U	ATCTCCGAAGTCTCGGCTTCGGAGCTTCATTGAGGG	36	19	Y	Y		
*B*. *asteroides*	PRL2011	I-E	GTGTTCCCCGCATCCGCGGGGATGATCC	28	147	Y	Y		
*B*. *biavatii*	DSM 23969	None	none						
*B*. *bifidum*	LMG 13200	II-A	GTTTCAGATGCCTGTCAGATCAATGACTTTGACCAC	36	23	Y		Y	
*B*. *bohemicum*	DSM 22767	I-C	GTCGCTCCCTTCACAGGGAGCGTGGATTGAAAT	33	20	Y	Y		
*B*. *bombi*	DSM 19703	II-C	CCAGTATATCAGAGGGGCTTTAGATTGAATTTGAAAC	37	25	Y		Y	
*B*. *boum*	LMG 10736	I-E	GTGTTCCCCGCGCATGCGGGGATGATCCC	29	157	Y	Y		
*B*. *breve*	UCC2003	I-C	GTCGATCCCCATCCGGGGAGCGTGGATTGAAAT	33	48	Y	Y		
*B*. *callitrichos*	DSM 23973	II-C	CAAGTCTATCAAGAAGGGTGAATGCTAATTCCCAAC	36	13	Y		Y	
*B*. *catenulatum*	DSM 16992	I-E	GTGTTCCCCGCATACGCGGGGATGATCCC	29	15	Y	Y		
*B*. *choerinum*	LMG 10510	Undetermined	GTGCTCCTCGCAAGCGCGTGGACAACCCG	29	20	N			
*B*. *coryneforme*	LMG 18911	None	none						
*B*. *crudilactis*	LMG 23609	I-C	GTCGCTCCCTCACGGGAGCGTGGATTGAAAT	31	36	Y	Y		
*B*. *cuniculi*	LMG 10738	I-E	AGTTGCCCCGCGTATGCGGGGATGATCCG	29	93	Y	Y		
*B*. *dentium*	LMG 11045	II-C	CAAGTTTATCAAGAAGGGTAGAAGCTAATTCCCAGT	36	17	Y		Y	
* *		I-C	GTCGCTCTCCTCACGGAGAGCGTGGATTGAAAT	33	81	Y	Y		
* *		Undetermined	TGTCCGATTCTCCAGAATCGGACA	24	8	N			
*B*. *gallicum*	DSM 20093	I-E	GTGCTCCCCGCAAGCGCGGGGATGATCC	28	39	Y	Y		
*B*. *gallinarum*	LMG 11586	None	none						
*B*. *indicum*	LMG 11587	None	none						
*B*. *kashiwanohense*	DSM 21854	None	none						
*B*. *longum subsp*. *infantis*	ATCC 15697	None	none						
*B*. *longum subsp*. *longum*	NCC2705	None	none						
*B*. *longum subsp*. *suis*	LMG 21814	Undetermined	GTCGCACCCCACTGGGGTGCGTGGATTGAAAT	32	9	N			
*B*. *magnum*	LMG 11591	Undetermined	GTGCTCCCCACATAGGTGGGGATGAT	26	4	N			
*B*. *merycicum*	LMG 11341	II-A	GTTTCAGATGCCTGTCAGATCAAGGACCTAGACCAC	36	87	Y		Y	
*B*. *minimum*	LMG 11592	I-E	GTTTGCCCCGCACTCGCGGGGATGATCC	29	149	Y	Y		
*B*. *mongoliense*	DSM 21395	None	none			N			
*B*. *moukalabense*	DSM 27321	Undetermined	ATTTCAATCCACGCTCTCCGTGAGGAGAGCGAC	33	15	Y			
*B*. *pseudocatenulatum*	DSM 20438	I-C	GTCGCTCTCCTCATGGAGAGCGTGGATTGAAAT	33	27	Y	Y		
*B*. *pseudolongum subsp*. *globosum*	LMG 11569	None	none						
*B*. *pseudolongum subsp*. *pseudolongum*	LMG 11571	I-E	GTTTGCCCCGCATGTGCGGGGATGATCCG	29	112	Y	Y		
*B*. *psychraerophilum*	LMG 21775	None	none						
*B*. *pullorum*	LMG 21816	I-U	ATTGCGAAGCTTTACGCTTCGCAACTTCATTGAGGA	36	20	Y	Y		
*B*. *reuteri*	DSM 23975	Undetermined	CCGAGGTTCCGCCCCGCTGAGGA	23	13	N			
*B*. *ruminantium*	LMG 21811	I-E	GTGTTCCCCGCATGCGCGGGGATGATCCC	29	67	Y	Y		
* *		Undetermined	ATGTCCGATTCTGCAGAATCGGACA	25	16	N			
*B*. *saeculare*	LMG 14934	None	none						
*B*. *saguini*	DSM 23967	I-C	GTCACCCTCCTCACGGAGGGTGCGGATTGAAAT	33	31	Y	Y		
*B*. *scardovii*	LMG 21589	I-E	GTTTACCCCGCATGCGCGGGGATGATCCG	29	66	Y	Y		
*B*. *stellenboschense*	DSM 23968	I-C	GTCGCCCCTCTCACGAGGGGCGTGGATTGAAAT	33	51	Y	Y		
*B*. *stercoris*	DSM 24849	Undetermined	TTGGATGTGAGCGGCTGGAACACC	24	6	N			
*B*. *subtile*	LMG 11597	I-C	GTCGCTCCCTCACGGGAGCGTGGATTGAAAT	31	156	Y	Y		
*B*. *thermacidophilum subsp*. *porcinum*	LMG 21689	Undetermined	TTGTGTGAGGATTTGCTCGCACA	23	5	N			
*B*. *thermacidophilum subsp*. *thermacidophilum*	LMG 21395	I-C	ATCGCTCCCCGTATGGGGAGCGTGAGTTGAAAT	33	89	Y	Y		
*B*. *thermophilum*	JCM 7027	I-U	ATTGCCGGGATTCAATTCCCGGCGCTTCATTGAGGG	36	53	Y	Y		
* *		Undetermined	GTCGCTCTCCTTACGGAGAGCGTGGATTGAAAT	33	11	N			
*B*. *tsurumiense*	JCM 13495T	I-U	ATTGCCAGAGTTATAAGCTCTGGCCTTCGTTGAGGA	36	7	Y	Y		
* *		II-C	CAATCTTATCAAGAGGGTAGAAAGCTAATTCACAGC	36	13	Y		Y	

Table 1 | Occurrence of CRISPR-Cas systems in bifidobacteria | Each strain used in this analysis was examined for CRISPR-Cas systems. “System Type” refers to the type and subtype of the predicted system. The sequence, repeat length, and number of repeats found in a single repeat-spacer array are given for each system. When repeat-spacer arrays were detected but no *cas* genes were annotated, the system type was designated “Undetermined.” “None” indicates that repeat-spacer arrays and *cas* genes were not found in the genome. *Cas* indicated the presence or absence of *cas* genes, with a “Y” indicating the gene is present and an “N” indicating the gene is absent. Type designation was confirmed by the presence of *cas3*, *cas9*, and *cas10* for Type I, II, and III respectively.

## Results

### Occurrence and diversity of CRISPR-Cas systems in *Bifidobacterium*

Among the 48 genomes analyzed, we observed a high rate of occurrence of CRISPR-Cas systems in the genus *Bifidobacterium* (77%) (Table 1), compared to the estimated prevalence in bacteria (45%, 1176/2612, as of the August 2014 update) [32]. In the 48 genomes analyzed, representing 43 species, we identified 26 Type I systems, and six Type II systems, while Type III systems appeared to be absent, according to the *cas* gene content and CRISPR repeat length and sequence. Interestingly, four strains contained multiple distinct CRISPR-Cas systems, including *B*. *dentium* which contained three separate systems. Each *Bifidobacterium* species containing a CRISPR-Cas system had a unique CRISPR repeat and *cas1* gene, suggesting that these systems are phylogenetically different from one another. Strains that contained repeat-spacer arrays but did not contain *cas* genes were categorized as “Undetermined”; given the repetitive nature of CRISPR loci and the draft genome status of many genomes, there are possibly additional loci that could not be identified or classified. *Bifidobacterium moukalabense* was also defined as “undetermined” because it only contained the repeat-spacer array and *cas1* and *cas2* genes; no other *cas* genes were found within this locus. The high rate of occurrence and occasional presence of multiple systems in a single genome suggests that bifidobacteria possess active CRISPR immune systems that are important in the defense against potentially damaging foreign DNA.

**Fig 1:**
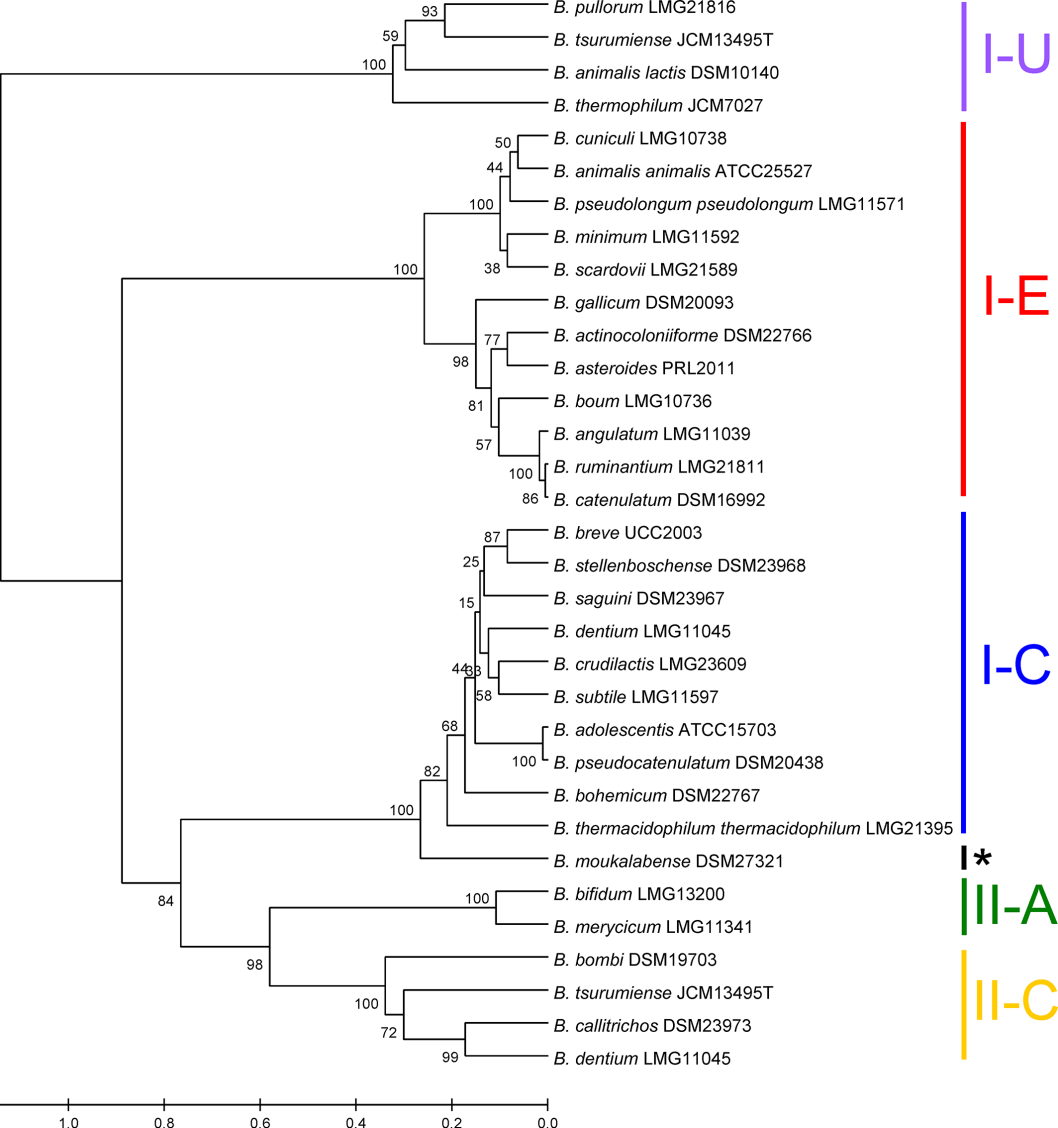
Clustering of Cas1 into distinct phylogenetic groups. Cas1 protein sequences were aligned using the MUSCLE algorithm and used to generate a UPMGA tree to show the divergence of different CRISPR-Cas systems. The system type and sub-type is noted on the right. The (*) indicates *B*. *moukalabense* which contained an “Undetermined” CRISPR-Cas system.

**Fig 2:**
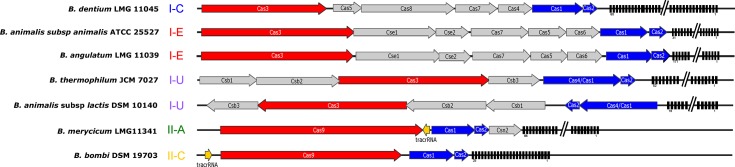
CRISPR-Cas locus architecture. One representative for each unique CRISPR subtypes represents the locus architecture of *cas* genes, CRISPR repeats, spacers and other system-specific components (e.g. tracrRNA). The signature gene for each subtype is colored in red (*cas3* or *cas9* for Type I and II, respectively). The universal *cas1* and *cas2* genes are colored in blue. Accessory genes are grey. The tracrRNA for Type II systems is shown in yellow. The direction of the arrows indicates directionality of the coding sequences. The repeat-spacer array only shows the CRISPR repeats (black rectangles). Each operon is shown at a scale of 11,000 base pairs. Long repeat-spacer arrays were shortened for simplicity indicated by a double line break. Numbers under the arrays indicate the first and last spacer location, showing the size of the array.

A phylogenetic analysis was performed to investigate the level of conservation and divergence between the identified bifidobacterial CRISPR immune systems using the universal Cas1 protein. The Cas1 tree in Fig 1 revealed five major clusters that correspond to different types and subtypes of CRISPR systems, namely a branch for Type I-E, I-C, I-U, Type II-A and II-C Cas1 proteins. Interestingly, the Type I-E branch appears to diverge further into two distinct sub-clusters that correspond to similar locus architectures but distinct *cas* genetic sequences (Fig 2). *B*. *moukalabense* was most likely a I-C system before loss of the other *cas* genes because it grouped most closely with the I-C branch. Finally, Type I-U systems appear to be the most distantly related Cas1s, diverging very early from the other Cas1 subtypes, including other Type I systems.

Type I systems, including the I-E, I-C, and I-U subtypes, were the most commonly encountered bifidobacterial CRISPR-Cas system, occurring at a rate of 54% (26/48), consistent with this system being also most prevalent in bacteria [13]. Ten systems were categorized as Type I-C based on a similar gene content and layout as *B*. *dentium* (Fig 2). Visible on the Cas1 tree (Fig 1), 12 species were split between two distinct Type I-E locus architectures: one cluster included *B*. *animalis* subsp. *animalis* and the other cluster included *Bifidobacterium angulatum*, with the main differentiating feature being the sequence of the *cas* genes, including the signature *cas3* and the universal *cas1* gene (Fig 2). Type I-U systems are very unique in that they contain a *cas4/*cas1 fusion gene. Three of the Type I-U systems matched this typical gene layout, as seen in *Bifidobacterium thermophilum*, whereas *B*. *animalis* subsp. *lactis* appears to have undergone a unique genomic rearrangement event of its Type I-U CRISPR-Cas system causing a reversed orientation of all relevant genes, thereby constituting a unique layout.

Type II systems, which are fairly rare in nature only occurring in 5% of bacteria [38], occur at a much higher frequency in the genus *Bifidobacterium*; Type II systems were found in six of the 48 genomes analyzed (12.5%), with two Type II-A systems and four Type II-C. The II-A *cas*9’s canonical arrangement and content are longer than the II-C *cas9*s; the II-A *B*. *merycicum cas9* is approximately 900 nucleotides longer than the Type II-C *cas9* in *B*. *bombi*. The predicted tracrRNA is located between *cas9* and *cas1* on the minus strand in *B*. *merycicum*, while the tracrRNA in *B*. *bombi*, a II-C system, is upstream of the 5’ end of *cas9* on the positive strand.

**Fig 3:**
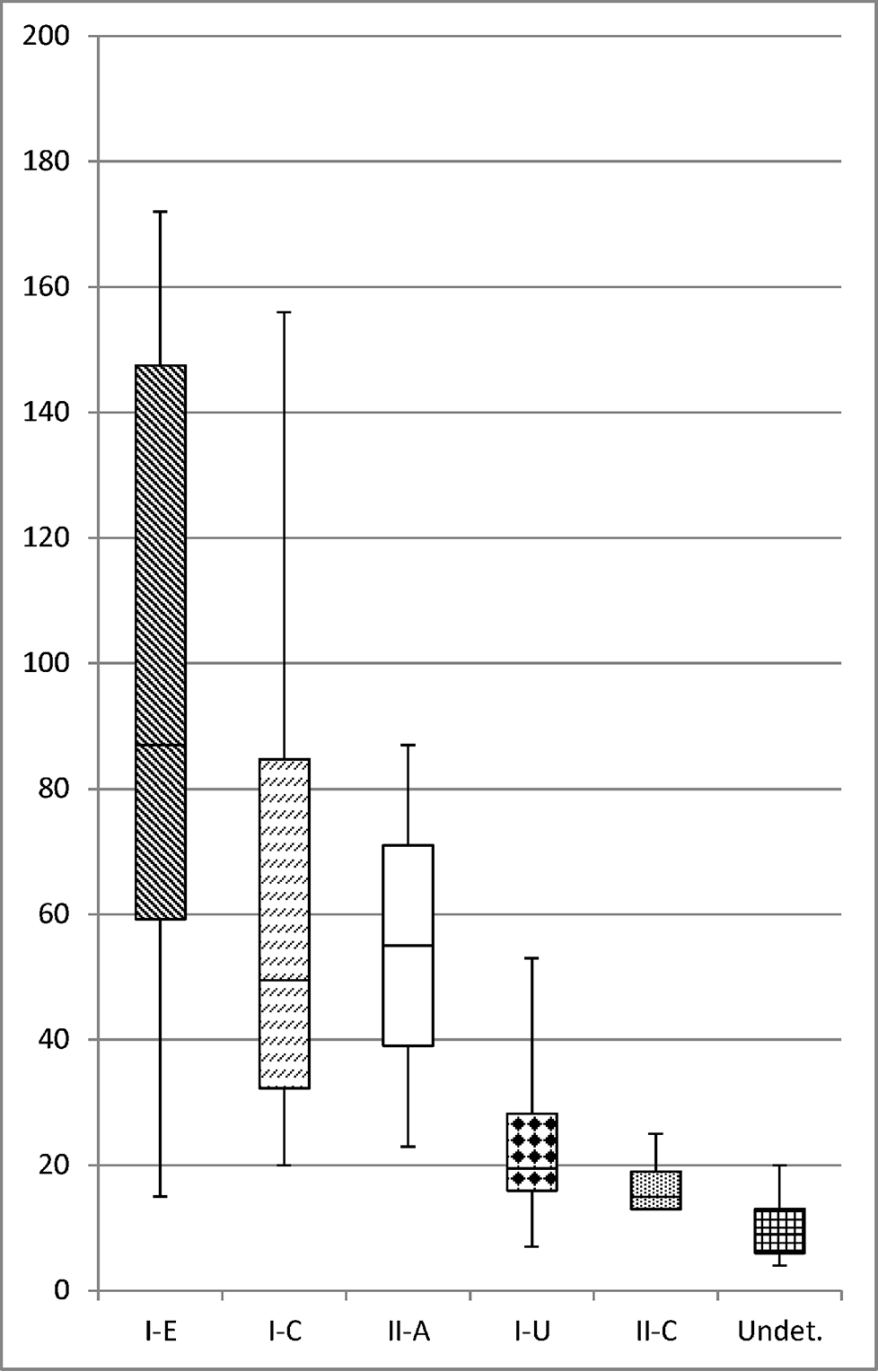
CRISPR repeat-spacer array size distribution. The graph shows the variability in size of the repeat-spacer arrays using number of spacers in each array, from Table 1. The error bars show the range of the locus size.

The size of the repeat-spacer arrays found in bifidobacteria is highly diverse, and ranging from 2 to 171 spacers (Fig 3, Table 1). Type I-E systems average 80 spacers per locus with some loci encoding as many as 171 unique genetic immunization events. Type I-C systems are, on average, smaller than Type I-E, averaging 50 spacers per locus, but can reach as many as 155 spacers. Type I-U systems average 19 spacers per locus with a range of 6 to 52 spacers. The identified bifidobacterial Type II-C systems contain, on average, the smallest repeat-spacer arrays (14 spacers). Because each unique spacer is acquired from invasive foreign genetic elements like phages and plasmids, we hypothesize that Type I-E systems are the most active loci as evidenced by their large size and highest rate of occurrence in the genomes. Such large arrays found in bifidobacteria also suggest these bacteria live in an environment rich in exogenic DNA requiring the need for an active defense system. Furthermore, such large repeat-spacer arrays could provide a locus with high genotyping potential. The hypervariable spacer sequences can be used to infer strain divergence and ecological relationships between different strains [7, 39].

### CRISPR spacer homology to prophage sequences

**Fig 4:**
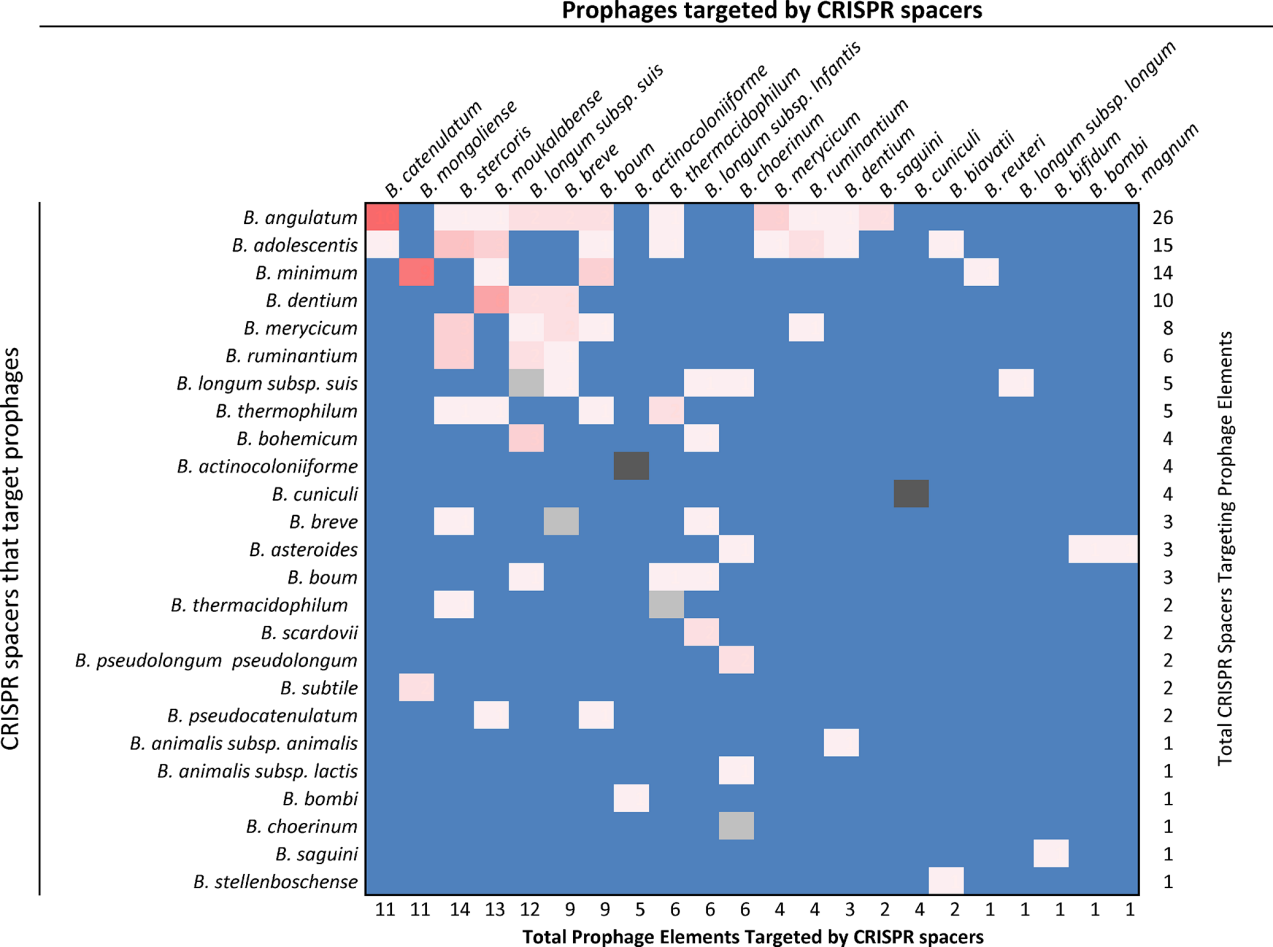
Prophage targeting by CRISPR spacers. The heat map displays *Bifidobacterium* CRISPR spacers that target prophage sequences harbored in bifidobacterial genomes. The horizontal axis lists hosts that contain prophages that are targeted by CRISPR spacers. The vertical axis lists strains containing CRISPR spacers that target a bifibacterial prophage sequence. The color intensity represents the number of cross-targeting events with red squares being high density (up to 10 targeting events), and white squares being single targeting events. The darker pink squares correlate with higher cross-targeting events and lighter pink squares correlate with fewer events. Blue squares represent that absence of CRISPR targeting. Hits shown in grey indicate self-targeting spacers that target prophage sequences in that particular chromosome meaning both the spacer and the prophage target are in the same chromosome. The darker grey indicates four distinct spacers that target prophage sequences, while the lighter grey indicates a single self-targeting spacer.

By investigating the foreign DNA source each spacer targets, insights can be gained as to the functional role of CRISPR-Cas systems. A protospacer is the DNA sequence homolog in a phage, plasmid, or chromosomal DNA that matches the spacer sequence stored in a CRISPR locus. Several matches to publically available metagenomic data and matches to prophage sequences in a local *Bifidobacterium* database (S1 Table), reveal that spacers in different genomes have homology to predicted (pro)phage genomes indicating bifidobacteria have acquired immunity against such (pro)phages (Fig 4).

The availability of 48 genome sequences of recognized bifidobacterial taxa [23, 29], allowed the identification of bifidobacterial prophage protospacers matching the spacer sequences of CRISPR loci identified in this study. Furthermore, as shown in S1 Table, investigation of flanking regions displaying a DNA similarity with the spacer sequences greater than 91% (e-value less that 0.0001), allowed us to precisely map their homologous region within the prophage regions identified in these bifidobacterial genomes.

The genomes of *B*. *angulatum*, *B*. *adolescentis* and *Bifidobacterium minimum* contain the most spacer sequences that target predicted prophages in bifidobacterial genomes (Fig 4). *B*. *angulatum* possesses 26 distinct spacers that target presumed prophage sequences in 11 different *Bifidobacterium* species. Conversely, the prophage sequences harbored in the genomes of *Bifidobacterium stercoris* and *B*. *moukalabense* taxa are potential targets of 14 and 13 CRISPR spacers, respectively. CRISPR systems that were shown to contain more than 10 spacers matching prophages in other genomes, did not contain prophages targeted by any other CRISPR systems. Conversely, genomes without CRISPR-Cas systems or that contained apparently degenerated CRISPR-Cas systems were more likely to have prophages that were targeted by other CRISPR systems. We speculate that one reason prophages may have been able to integrate into host chromosomes is the lack of an interfering CRISPR-Cas system. There is a correlation between genomes targeted by CRISPR spacers and absence of functional *cas* genes. However, no one type of CRISPR-Cas systems was correlated with higher numbers of spacers targeting prophages.

CRISPR-Cas systems defined as “undetermined” and degenerate rarely had spacer sequences that target prophages; only *B*. *longum* subsp. *suis* had four spacer sequences that showed homology to prophage sequences (Fig 4). Of the 32 characterized and putative functional CRISPR-Cas systems in the genus *Bifidobacterium*, 25 had at least one spacer sequence that targeted a prophage. The most frequently targeted prophage-containing species are *B*. *longum* subsp. *suis*, *Bifidobacterium catenulatum* and *Bifidobacterium mongoliense* which range from 11 to 12 protospacer targets in their prophage regions (Fig 4). Interestingly, *Bifidobacterium actinocoloniiforme* and *Bifidobacterium cuniculi* display some spacer matches against their own prophage sequences and none against other bifidobacterial taxa. Self-targeting spacers suggest that those CRISPR-Cas systems may be inactive to prevent Cas protein cleavage of the chromosome, as self-targeting is known to be a lethal event [40].

### Characterization of Type II CRISPR-Cas elements

**Fig 5:**
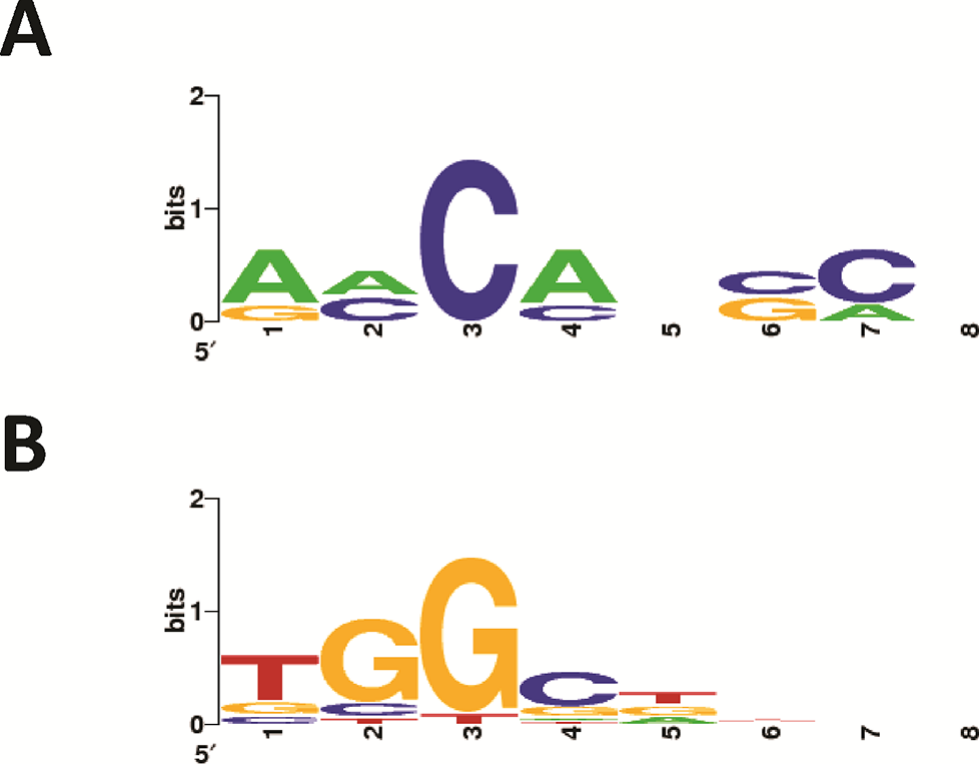
WebLogo predictions of PAMs. The height of each letter represents the conservation of that nucleotide at each position in the 10nt flank at the 3’ end of the protospacer. Hits from S1 Table were used to generate these WebLogos.

**Fig 6:**
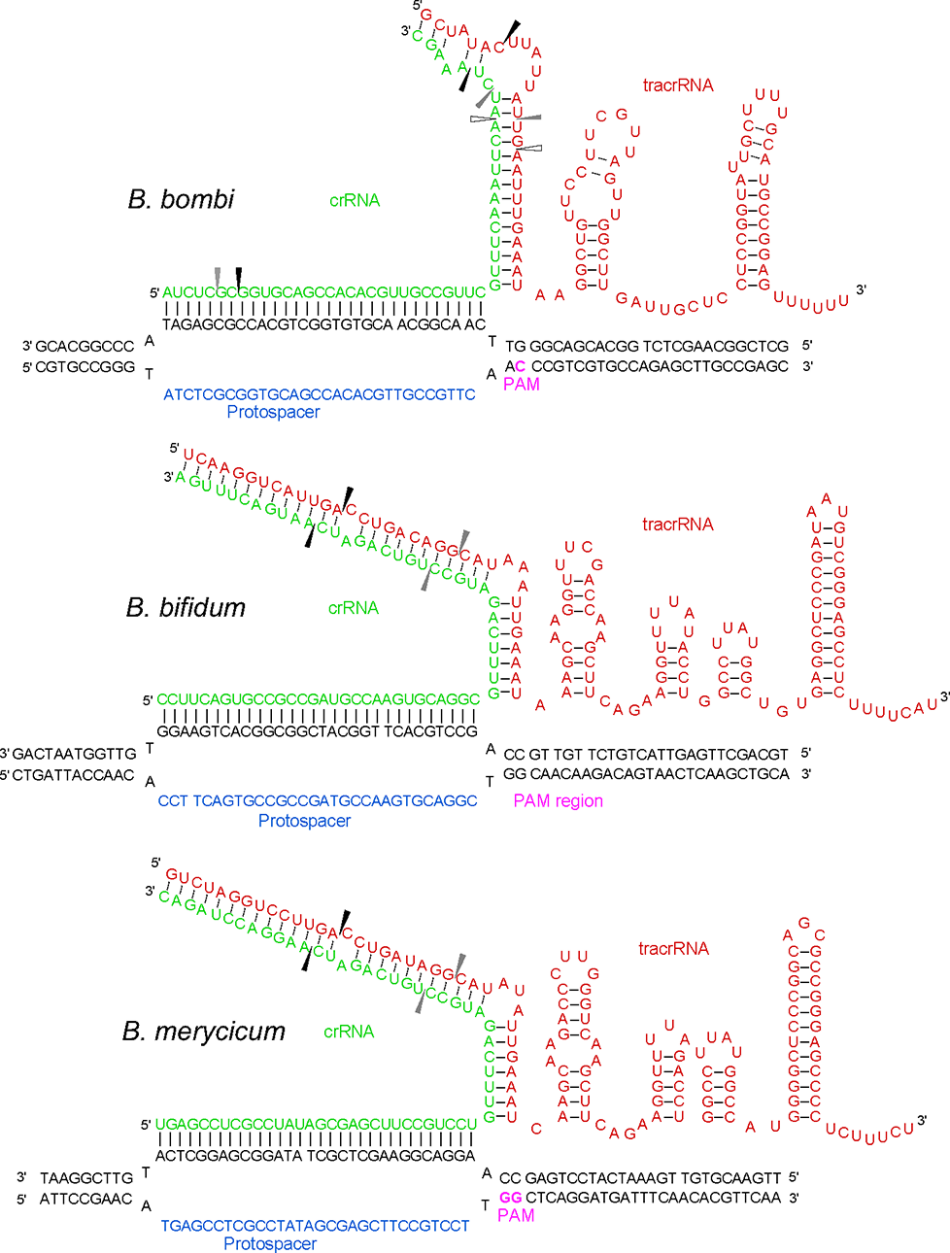
crRNA:tracrRNA duplex binds with target DNA sequence next to PAM sequence. Type II elements involved in Cas9 targeting and cleavage for *B*. *bombi* (A), *B*. *bifidum* (B), and *B*. *merycicum* (C) include the target protospacer (blue), the recognition PAM (purple), crRNA (green), and tracrRNA (red). The tracrRNA:crRNA duplex come together and induce endonucleolytic activity of Cas9 to cleave foreign DNA. The PAM was not able to be determined for *B*. *bifidum*, so the PAM region on the target DNA strand is shown instead. The RNaseIII processing sites were inferred from the preliminary RNASeq data and previous characterization of RNaseIII activity [41, 42]; the darkest arrow is most likely the primary processing site and the lighter arrows are the secondary and tertiary processing sites. For *B*. *bombi*, the processing sites were based on the boundaries determined for the crRNAs using the RNASeq data. For *B*. *bifidum*, the processing sites were determined using the boundaries from the tracrRNA RNASeq data. The *B*. *merycicum* sites are based on the boundaries from the *B*. *bifidum* data; the tracrRNA sequences only differed by five nucleotides in the upperstem-bulge-lowerstem region, meaning the processing sites are likely similar.

To fully characterize the Type II CRISPR-Cas systems in three *Bifidobacterium* genomes, we identified the tracrRNA sequence, predicted the structure of the crRNA:tracrRNA duplex, and investigated the PAM sequence targeted by Cas9 by identifying protospacers for the spacer sequences in these genomes (Figs 5 and 6, S1 Table). Three representative organisms were selected for further characterization: *B*. *merycicum* and *B*. *bifidum* which contain Type II-A systems and *B*. *bombi* which contains a II-C system.

The tracrRNAs predicted for the three organisms are all congruent with conserved tracrRNA: crRNA structures as previously published in the literature [36, 38], and contain a double stemmed nexus previously only seen in lactobacilli. Combining preliminary RNASeq analyses (S1 Text) and predicting RNase III processing sites as defined by Pertzev and Nicholson, the boundaries of the crRNA suggest that the 5’ processing site occurs either just below the bulge at the top of the lower stem, or after the first nucleotide of the upper stem, in the crRNA:tracrRNA modules previously defined by Briner et al., 2014 [36, 41, 42] (Figs 5 and 6, S1 Fig). For both II-A systems, *B*. *bifidum* and *B*. *merycicum*, the crRNA:tracrRNA duplexes appear to have one processing site in the middle of the upper stem and a second processing site one to two nucleotides after the bulge; these similar processing sites are likely due to similarity of crRNA and tracrRNA sequences [36, 41, 42].

Boundaries for the crRNA and tracrRNA molecules are important for characterizing how Type II CRISPR-Cas systems function, but the protospacer adjacent motif (PAM) is another element that is important for functionality; the PAM is a recognition motif used by the Cas proteins to bind foreign DNA (Fig 5) [10–11,43]. In Type II-A systems, Cas9 first binds to the PAM and then interrogates the DNA from 3’ to 5’ [44]. The PAM identified for *B*. *merycicum*, 5’-NGG-3’, is the same as the well-characterized *Streptococus pyogenes* PAM, and flanks the 3’ edge of the protospacer (Figs 4 and 5, S1 Table). Interestingly, the PAM identified for *B*. *bombi*, a Type II-C system, is predicted to be a single nucleotide 5’-NNG-3’ flanking the 3’ end of protospacers. Altogether, using the PAM, tracrRNA, CRISPR repeat, and novel Cas9, Type II systems can be exploited to develop new genome editing tools [17, 19].

## Discussion

Bifidobacteria have very frequent, diverse and large CRISPR-Cas systems. The large repeat-spacer arrays provide a hypervariable, yet conserved, loci that can be used for genotyping strains. Such large and diverse CRISPR-Cas loci indicate these systems are likely active and important in bifidobacterial survival and evolutionary relatedness. While we were able to distinguish five unique subtypes of CRISPR-Cas systems in *Bifidobacterium*, a previous study by Horvath *et al*. comparing CRISPR-Cas systems in lactic acid bacteria proposed the genus only spanned three distinct subtypes [45]. By using a larger data set, we were able to show that the CRISPR systems in *Bifidobacterium* are more diverse than previously reported and include Type I-U and II-A systems that had previously not been identified in bifidobacteria. Surprisingly, the strain of *B*. *longum* used in the Hovarth et al. study contained a Type II-C system, but among the three subspecies of *B*. *longum* used in this study (*B*. *longum* subsp. *infantis*, *B*. *longum* subsp. *longum*, and *B*. *longum* subsp. *suis*), only a remnant of a CRISPR-Cas system was detected in *B*. *longum* subsp *suis* [45]. This finding suggests that the presence of CRISPR-Cas systems in *Bifidobacterium* may be strain- rather than species-dependent, complicating estimations of the exact prevalence of these systems across the entire genus.

Spacer sequences that showed positive matches to various phage and prophage DNA in genomic and metagenomic data suggest that *Bifidobacterium* live in (pro)phage-rich environments and require defense systems capable of adapting to predation from phages. Interestingly, there was a correlation between the presence of CRISPR-Cas system and the number of CRISPR spacers that were able to target prophage sequences in other *Bifidobacterium* genomes. Some bifidobacteria strains display a high number of protospacer matches to other bifidobacterial-specific prophages integrated in the genomes of other *Bifidobacterium* species. In this context, *B*. *angulatum* has 10 CRISPR spacers matching *B*. *catenulatum* prophages, *B*. *dentium* has six spacers that target *B*. *moukalabense* prophages, and *B*. *minimum* has nine spacers that target *B*. *mongoliense*. Such findings might be explained by these organisms inhabiting same ecological niches followed by co-evolution with a similar repertoire of phages. Futhermore, as described in the recent work aimed to reassess the bifidobacterial taxonomy [29], *B*. *dentium* and *B*. *moukalabense* are two of the eight bifidobacterial species fitting in the *B*. *adolescentis* group. The identification of those shared spacer sequence targets further supports close evolutionary development of these two genomes and may suggest CRISPR spacer conferred prophage exclusion that differentiated the *Bifidobacterium* genus [29].

Preliminary transcriptome data suggests some of these systems are actively being transcribed and maintained, supporting the hypothesis that attack from (pro)phages is an ever-present threat to *Bifidobacterium*. This machinery that naturally targets foreign DNA from Type II CRISPR-Cas systems can be exploited to create new tools for genome editing and engineering. *B*. *bombi* may be an intriguing new prospect for development of new tools as the predicted PAM is only a single nucleotide and the Cas9 protein (1239 amino acids) is 129 amino acid residues smaller than the widespread *S*. *pyogenes* Cas9 (1368 amino acids) [19].

Altogether, the bifidobacterial CRISPR-Cas systems constitute a framework for various potential biotechnological and ecological uses. The frequent prevalence of large and diverse CRISPR loci provides a platform for strain genotyping and differential evolutionary studies. CRISPR spacer sequences can help provide insights into historical phage-host interplay as well as broaden our understanding of phage resistance in *Bifidobacterium*. The characterization of Type II elements in a select set of bifidobacteria may also open the door for next generation molecular tools for genome editing in bacteria and potentially eukaryotes.

## Supporting Information

S1 Fig Locus transcriptional profiles. (A) small RNA molecules from *B*. *bombi* that mapped to the CRISPR repeat-spacer array. (B) boundaries of spacer ten, the most highly transcribed spacer in the *B*. *bombi* CRISPR repeat-spacer array. Other show RNA reads that mapped to the CRISPR-Cas system in *B*. *bifidum*. (C) transcripts mapped to the entire locus suggesting this system is transcribed. (D) transcripts that map to the tracrRNA sequence; the 5’ boundaries for this molecule can be determined.
(TIFF)

S1 Table Protospacer hits used to determine the PAM sequence. (DOCX)

S1 Text Preliminary RNASeq methods and analysis. Materials and methods are provided for RNASeq analyses carried out to assess CRISPR-Cas locus transcription, and determine guide RNA sequences.
(DOCX)
