# Supplementary figures and images for: Occurrence and Diversity of CRISPR-Cas Systems in the Genus Bifidobacterium
Source: PLoS One. 2015 Jul 31;10(7):e0133661. doi: 10.1371/journal.pone.0133661 (PMC4521832; doi:10.1371/journal.pone.0133661)

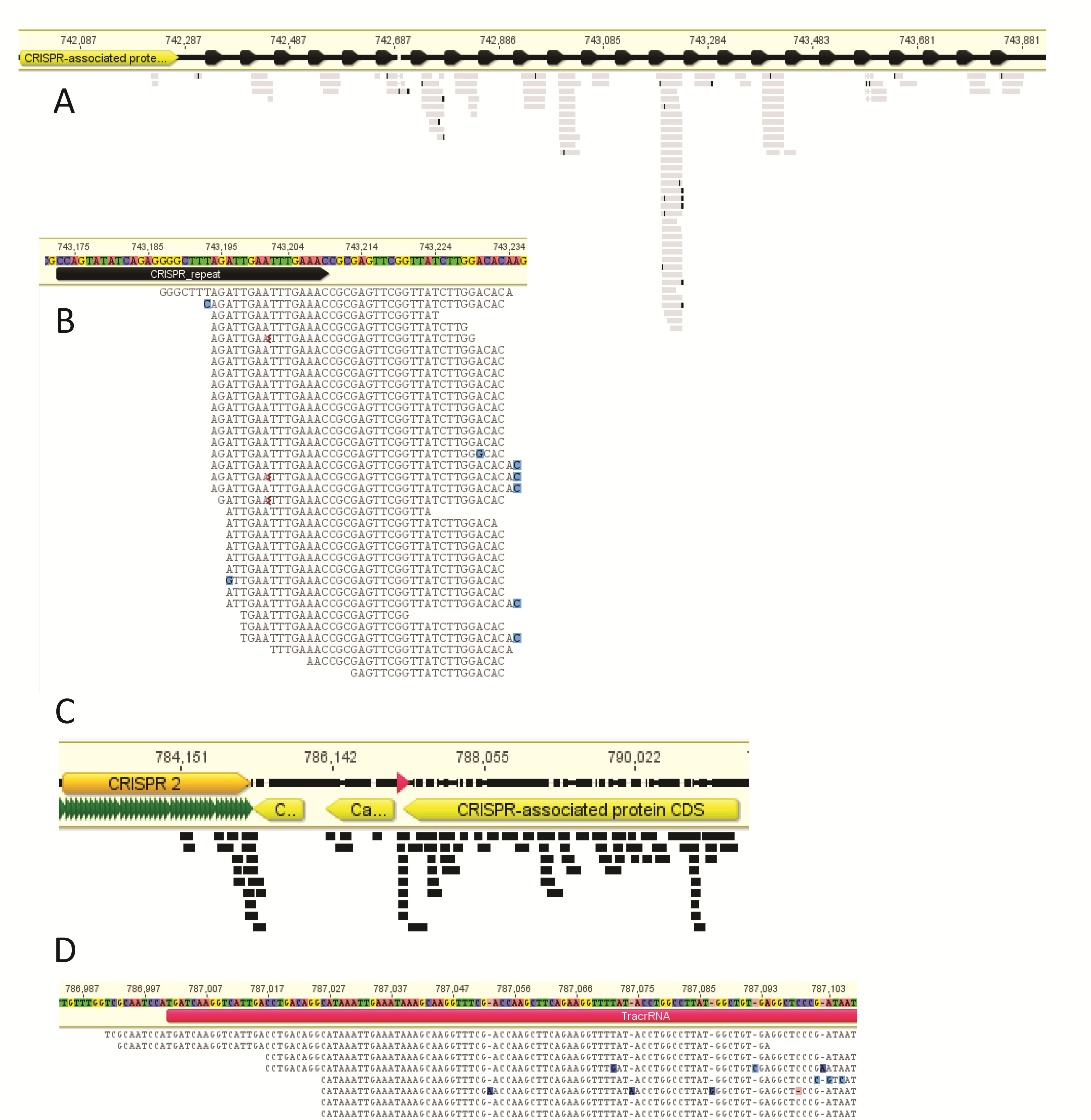

Supplement: S1 Fig — (A) small RNA molecules from B. bombi that mapped to the CRISPR repeat-spacer array. (B) boundaries of spacer ten, the most highly transcribed spacer in the B. bombi CRISPR repeat-spacer array. Other show RNA reads that mapped to the CRISPR-Cas system in B. bifidum. (C) transcripts mapped to the entire locus suggesting this system is transcribed. (D) transcripts that map to the tracrRNA sequence; the 5’ boundaries for this molecule can be determined. (TIFF) [file pone.0133661.s001.tiff]
