# Supplementary material for: Occurrence and Diversity of CRISPR-Cas Systems in the Genus Bifidobacterium
Source: PLoS One. 2015 Jul 31;10(7):e0133661. doi: 10.1371/journal.pone.0133661 (PMC4521832; doi:10.1371/journal.pone.0133661)
Supplement: S1 Table — (DOCX) [file pone.0133661.s002.docx]

Table S1 | Protospacer hits used to determine the PAM sequence

|  | ***Bifidobacterium bombi*** |  |  |  |  |  |  |
| --- | --- | --- | --- | --- | --- | --- | --- |
| Spacer No. | Protospacer match | Accession No. of Match | Location | Left Flank | Protospacer sequence | Right Flank | Match Coverage |
| 4 | Food metagenome | AEKF01000049.1 | 13048 to 13095 | CCTTCTTGCA | TACGTGCGCCACCTGATGCAGTGAACAA | AACAACCGGT | 24/28 |
| 6 | Uncultured *Bifidobacterium* | ADJS01003436.1 | 376 to 423. | GAACATCGAA | AACGCGCAGGCCAAACTCAAAGGCCTCG | GCCACGACTC | 25/28 |
| 4 | *L. mesenteroides* acetyl-coA carboxyl transferase | JAUJ01000006.1 | 114144 to 114191. | CCTTCTTGCA | TACGTGCGCCACCTGATGCAGTGAACAA | AACAACCGGT | 24/28 |
| 21 | *Bifidobacterium breve* | CP006715.1 | 374835 to 374882. | GACGGGTTGA | ACGGCAACGTGTGGCTGCACCGCGAGAT | ACCCGGCACG | 25/28 |
|  |  |  |  |  |  |  |  |
|  | ***Bifidobacterium merycicum*** | |  |  |  |  |  |
| Spacer No. | Protospacer match | Accession No. of Match | Location | Left Flank | Protospacer sequence | Right Flank | Match Coverage |
| 23 | *Bifidobacterium boum* | JHWO01000002.1 | 27282 to 27331. | GGAGACACAG | AACAAGGTGGCCGAGGCCATCTACCTGCGC | TGGTATAGCA | 30/30 |
| 42 | Mycobacterium phage major tail subunit | ADJS01003585.1 | 82388 to 82437. | ATTCCGAACA | TGAGCCTCGCCTATAGCGAGCTTCCGTCCT | TGGCTCAGGA | 30/30 |
| 48 | *Bifidobacterium ruminantium* | JHWQ01000014.1 | 52317 to 52366. | TGCTACACCC | ACCCCGCGGTGTATGAGGCGATAAAGGACT | GGGCATGCCG | 27/30 |
| 50 | *Corynebacterium* phage HNH endonuclease | JHWQ01000014.1 | 28727 to 28776. | ACGATACGGC | CTGACCGTATCACGCAACCTCATCAGCCAA | TGGCTCAAAC | 30/30 |
| 50 | *Bifidobacterium breve* | AFXX01000010.1 | 47903 to 47952. | ACGATACGGC | CTGACCATATCACGCAACCTCATCAGCCAA | TGGCTCAAAC | 30/30 |
| 56 | Phage integrase family protein | ADJS01003586.1 | 981 to 1030. | GTTCGCGTAT | GAGGTGACTGTCGGAGAGAGTGAGGATACA | TGGCGTCTCG | 28/30 |
| 57 | Uncultured *Bifidobacterium* sp. | ADJS01003585.1 | 81246 to 81295. | GTATGCGAAC | CGTGAGCGTGAACGTTAACGTTGATACGGT | CGGCGACACT | 28/30 |
| 59 | *Bifidobacterium pseudocatenulatum* | CBEV010000166.1 | 2341 to 2390. | CGTCCGGCCT | TGACCAGACGGGTGTACATGCGGACCGCTG | CGGCATGATT | 28/30 |
| 60 | *Bifidobacterium breve* | AFXX01000010.1 | 46087 to 46136. | GAGCGCTTGC | AGGACGTGTTCGCATTGCCACGTGTGTATG | TGGCGTTTCG | 29/30 |
| 76 | *Bifidobacterium pseudocatenulatum* phage capsid protein | JEOD01000016.1 | 127988 to 128037. | TTCTTGGTCG | TGGTGGTGCTCGGCGAGTATTCCGTGTTGA | TGGCGCGGAA | 30/30 |
