# Supplementary material for: Occurrence and Diversity of CRISPR-Cas Systems in the Genus Bifidobacterium
Source: PLoS One. 2015 Jul 31;10(7):e0133661. doi: 10.1371/journal.pone.0133661 (PMC4521832; doi:10.1371/journal.pone.0133661)
Supplement: S1 Text — Materials and methods are provided for RNASeq analyses carried out to assess CRISPR-Cas locus transcription, and determine guide RNA sequences. (DOCX) [file pone.0133661.s003.docx]

**Supporting Information.**

**S1 Text. Preliminary RNASeq methods and analysis.** Materials and methods are provided for RNASeq analyses carried out to assess CRISPR-Cas locus transcription, and determine guide RNA sequences.

**RNASeq Methods**

Total RNA was isolated using previously described methods [31]. One hundred ng of total RNA was used as the starting input for RNA-Seq library preparation. Briefly, 100 ng of total RNA was treated with MICROB*Express*^TM^ Bacterial RNA Enrichment Kit (Ambion) to remove rRNA according to supplier’s instructions. The yield of rRNA depletion was checked by Experion (BioRad, UK). Then, rRNA-depleted RNA samples were fragmented using RNaseIII (Life Technologies, USA) followed by size evaluation using the TapeStation (Agilent, USA). Whole transcriptome libraries were constructed using the Ion Total-RNA Seq Kit v2 (Life Technologies, USA). Barcoded libraries were quantified by qRT-PCR and each library template was amplified on Ion Sphere Particles using Ion One Touch 200 Template Kit v2 (Life Technologies, USA). The samples were loaded on 316 Chips and sequenced by means of a PGM instrument (Life Technologies, USA).The RNASeq data for *B. bifidum* consist of 200 base pair, single strand reads. For *B. bombi* the small RNAs from 10 to 40 nucleotides were preserved using the Total RNA-seq kit v2 (Ion Torrent) kit following the Small RNA protocol. The reads were processed, mapped, and visualized in Geneious® 7 (Biomatters). The reads were filtered by length, then trimmed at the ends, keeping nucleotides above a 90% confidence interval (Q=10), and mapped to the *B. bifidum* IF 23 or *B. bombi* DSM 19703 genome using Bowtie 2. For *B. bifidum,* reads that were 100 to 250 nucleotides in length were kept; for *B. bombi* reads with a length between 5 and 100 nucleotides were retained.

**RNASeq Analysis**

In order to investigate whether the identified bifidobacterial CRISPR-Cas systems are active, transcriptome data was generated for two different organisms. For *Bifidobacterium bombi* DSM 19703, which contains a Type II-C CRISPR-Cas system, we extracted only small RNA molecules less than 40 nucleotides in length to look for transcripts of the crRNAs. We were able to identify RNA reads that mapped to CRISPR spacers 8 through 24 in the Type II-C locus in DSM 19703 strain (Supplemental Figure 2), suggesting that this locus is actively transcribed and provides protection against invasive elements, such as prophages. In this analysis, we observed that the middle of the locus was more highly transcribed than the leader end of the array, which has been reported previously [35, 36]. However, as our study was not a quantitative analysis for transcription levels, this finding may be due to library preparation, sequencing bias, quality assurance parameters used to trim and filter the reads, or parameters used to map the reads.

For *B. bifidum,* which contains a Type II-A system, instead of preserving the small RNAs, a typical RNA extraction protocol was performed, and transcriptome data from four expression analyses were pooled. Because small RNAs would have been lost in this procedure, no crRNA reads were detected. However, we were able to identify transcription of all *cas* genes as well as the predicted tracrRNA molecule (Supplemental Figure 2).
